# Evaluation of Patient-Reported Outcome Measures (PROMs) Associated With the Acceleration of Canine Retraction by Piezosurgery in Comparison With Low-Level Laser Therapy: A Three-Arm Randomized Controlled Clinical Trial

**DOI:** 10.7759/cureus.51779

**Published:** 2024-01-07

**Authors:** Doa'a Tahseen Alfailany, Mohammad Y. Hajeer, Mohammed A. Awawdeh, Mohammad Khursheed Alam, Khaldoun M.A. Darwich, Ossama Aljabban, Youssef Latifeh, Jacqueline Bashar Alhaffar, Imad Addin Almasri

**Affiliations:** 1 Department of Orthodontics, Faculty of Dentistry, University of Damascus, Damascus, SYR; 2 Department of Preventive Dental Science, College of Dentistry, King Saud Bin Abdulaziz University for Health Sciences, Riyadh, SAU; 3 Department of Preventive Dental Science, College of Dentistry, Jouf University, Sakaka, SAU; 4 Department of Oral and Maxillofacial Surgery, Faculty of Dentistry, University of Damascus, Damascus, SYR; 5 Department of Endodontics and Restorative Dentistry, Faculty of Dentistry, University of Damascus, Damascus, SYR; 6 Department of Internal Medicine, Faculty of Medicine, University of Damascus, Damascus, SYR; 7 Department of Applied Statistics, Faculty of Economics, University of Damascus, Damascus, SYR

**Keywords:** satisfaction, patient-reported outcome measures, low-level laser therapy, piezocision, discomfort, pain, canine retraction

## Abstract

Background and objectives

Recently, both surgical and non-surgical interventions have gained popularity in accelerating orthodontic tooth movement, but there is no randomized controlled trial (RCT) comparing both modalities in terms of patient-reported outcome measures (PROMs) during maxillary canine retraction. Therefore, this trial aimed to assess the PROMs associated with either low-level laser therapy (LLLT) or piezocision-assisted acceleration in the context of maxillary canine retraction.

Materials and methods

This was a single-blinded, single-center, three-arm RCT. A total of 54 patients (12 males, 42 females, mean age 20.65 ± 2.85) whose treatment needed upper-first-premolar extraction to facilitate canine retraction were enrolled and randomly divided into three groups: piezocision group (PG), LLLT group (LLLTG), and the control group (CG). Standardized questionnaires using a visual analog scale were distributed to patients at five assessment times: 1 (T1), 3 (T2), 7 (T3), 14 (T4), and 28 days following the canine retraction initiation (T5). The patients’ pain, discomfort, swelling, chewing difficulty, satisfaction, and acceptance were recorded.

Results

Regarding pain and discomfort, the levels were significantly lower in the LLLTG during the first two weeks of canine retraction compared to the other two groups (p<0.017). At the same time, these levels were significantly greater in the PG than the CG in the first week of canine retraction (p<0.017). Patients in the PG had a "mild to moderate" perception of swelling at T1 and T2, which was significantly different than that of the other two groups (p<0.001). Regarding chewing difficulty, the levels in the LLLTG were significantly lower than those in PG at the first three assessment times (p<0.017). Patients’ satisfaction with canine speed was significantly greater in the intervention groups compared to the CG (p<0.001). In contrast, no statistically significant differences were found between the three groups regarding satisfaction with gum appearance surrounding the canine (p=0.061) and acceptance (p=0.125).

Conclusion

The LLLT-assisted canine retraction was associated with significantly lower negative patient-reported outcomes during the first two weeks of retraction than piezocision-assisted retraction. However, the levels of pain and discomfort were significantly greater in the piezocision-assisted retraction group than those in the conventional canine retraction group, which in turn were greater than those with the LLLT-assisted canine retraction group during the first week of retraction. Patient satisfaction and acceptance were high with both piezocision and LLLT interventions.

## Introduction

In recent decades, the trend has increased towards accelerating the orthodontic tooth movement (OTM) to shorten the treatment duration and eliminate the side effects associated with prolonged treatment [1]. In addition to fulfilling the requirements of patients to end orthodontic treatment in the shortest period for aesthetic and social reasons [2,3]. Several acceleration methods have been presented, which can be divided according to their invasiveness into conservative non-surgical acceleration methods (biomechanical, pharmacological, and physical techniques) and surgical ones [4].

Low-level laser therapy (LLLT) is a promising physical acceleration method that has improved orthodontic treatment by decreasing treatment time through its photobiomodulation impact and by modifying tooth movement pain through its analgesic and anti-inflammatory impacts [5,6]. The exact mechanism of the LLLT analgesic effect is still unclear. However, some studies have indicated reduced nerve fiber activity after LLLT [7], while other studies indicate that LLLT reduces inflammatory mediators, alters nerve impulse transmission, and releases endorphins [8].

On the other hand, surgical methods are among the most acceleration methods in application and testing, with promising results in accelerating the OTM [9]. Piezocision is one of the minimally invasive surgical techniques that has gained more prominence in the literature [10]. Piezocision is a promising technique for accelerating OTM due to its various orthodontic, aesthetic, and periodontal advantages [11]. Despite the success of the piezocision technique in accelerating OTM, there is a lack of evidence regarding the acceptability, quality of life, and pain and discomfort associated with this technique [12].

After reviewing the literature, previous findings regarding pain associated with LLLT-assisted accelerated maxillary canine retraction have been controversial. Some studies found that the laser reduces the pain associated with accelerating canine retraction [13-15], while some indicate that LLLT was ineffective [16,17]. Moreover, a very recent Cochrane systematic review concluded that the evidence supporting the LLLT as a method of pain control by fixed appliances during orthodontic treatment was insufficient [18].

On the other hand, patient-reported outcome measures (PROMs) accompanying piezocision suffer from a lack of strong evidence to support it and need more high-quality randomized controlled trials (RCTs) [19]. Additionally, previous studies that investigated PROMs associated with either LLLT or piezocision-assisted acceleration in the context of canine retraction had a split-mouth design, and this design of studies, according to Mousa et al.'s systematic review, may confuse patients in their assessment of pain. Moreover, assessing pain on one side of the mouth does not reflect the full picture of pain and discomfort for such techniques proposed to be applied bilaterally [19].

To date, there has been no clinical study that has compared PROMs associated with the application of either piezocision or LLLT. In a recent systematic review, Alfailany et al. emphasized the need for studies comparing surgical and non-surgical methods concerning PROMs and associated side effects [20]. Therefore, this study was carried out to assess, comparatively, the PROMs associated with either LLLT or piezocision-assisted acceleration in the context of maxillary canine retraction.

## Materials and methods

Study design, registration, and settings

This trial was a single-centered, three-arm, parallel-group RCT performed at the Orthodontics Department, Damascus Dental University. However, the study was registered at the database of ClinicalTrials.gov (ID: NCT05265416). The approval of the Research Ethics Committee was obtained (UDDS-822-28102020/SRC-5521), and the funding was received from the Postgraduate Research Budget of Damascus Dental University (Ref no: 82583005182DEN).

Sample size calculation

Minitab® software (version 20.4; Minitab Inc., State College, PA) was adopted to calculate the sample size. The calculation was conducted using a 5% alpha level and 85% test power with the assumption that the least significant difference to be detected between groups in pain level is 25 mm on the visual analog scale (VAS) with a standard deviation of 23.4 (from a previous paper [21]). Applying the previous assumptions with a one-way ANOVA, it was found that 17 patients were required in each group. However, the number was increased, assuming that withdrawal might occur during the follow-up. Therefore, the total number required for the sample was 54 (i.e., 18 patients in each group).

Patients' recruitment and eligibility criteria

Patients were screened in the Orthodontics Department at the Faculty of Dentistry at Damascus University between October 2021 and March 2022. However, the patients who agreed with the inclusion criteria were presented with adequate information about the research, and informed consent was obtained from them. During case selection, the following inclusion criteria were taken into account: (1) adult healthy male and female patients, (2) age range of 17-28 years, (3) mild-to-moderate skeletal Class II (5 ≤ skeletal relationship in the median plane (ANB) ≤7) with overjet ≤10, (4) normal or excessive facial height (which was assessed clinically and then cephalometrically using these three angles: mandibular/cranial base angle, maxillary/mandibular plane angle, and facial axis angle), (5) the crowding of maxillary anterior teeth ≤ 4, and (6) periodontally sound dentition, which was judged clinically (probing depth < 4 mm, gingival index ≤ 1, plaque index ≤ 1) [22]. Exclusion criteria were as follows: (1) medical problems that affect OTM (corticosteroid, osteoporosis, hyperparathyroidism, and uncontrolled diabetes), (2) presence of a medical contraindication to anesthesia and surgical procedure, (3) loss of any of the maxillary permanent teeth (except third molars), and (4) previous orthodontic treatment history [23].

Randomization, allocation concealment, and blinding

Block randomization was conducted by distributing the patients participating in the research across nine blocks so that each block included six patients with a 1:1:1 allocation ratio. For each block, a random numbers list was produced by an orthodontist unrelated to this trial using Minitab® software (version 20.4; Minitab Inc., State College, PA). The patients were randomly assigned to three groups: the first group received canine retraction assisted by piezocision (PG), the second one received canine retraction assisted by LLLT (LLLTG), and the third group underwent conventional canine retraction (CG). The random sequences were hidden in opaque, closed envelopes, which were unsealed only after the leveling and alignment phase ended. Blinding either the clinician or patients was not possible. However, the principal researcher (DTA) who performed the measurements was ignorant of treatment assignments for included patients. Moreover, before processing and analysis, all data were coded, which guarantees the blinding of this trial stage. 

Orthodontic procedures

All patients of the three groups underwent fixed orthodontic treatment by the principal investigator (DTA) under the oversight of the coauthor (MYH) at the Orthodontic Department. At first, the patient was referred to the Oral and Maxillofacial Surgery Department to extract the first upper premolars. Thereafter, the teeth were aligned and leveled using MBT 0.022-inch brackets (Votion™, Ortho Technology®, FL), and the wire sequence was followed up until reaching a 0.019 x 0.025-inch stainless steel (SS) wire (JISCOP, Gunpo-si, Gyeonggi-do, Korea), which was considered the basal archwire. However, to secure a moderate anchorage, welded trans-palatal arches were applied since the beginning of the treatment.

Acceleration procedures

Piezocision Surgical Procedure

**Figure 1:**
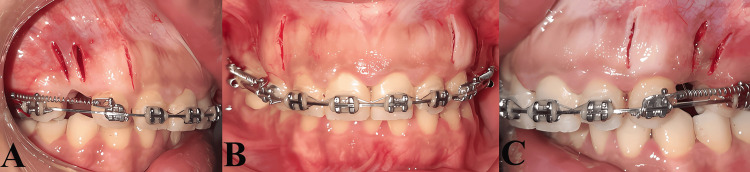
The piezocision procedure: A, the right buccal view; B, the frontal view; and C, the left buccal view

The flapless piezocision was carried out by the surgeon (NA) under the supervision of one of the coauthors (BB) at the Department of Oral and Maxillofacial Surgery of Damascus Dental University. Before the commencement of the surgical intervention, the patients were instructed to rinse with chlorhexidine 0.12% for a minute. Following the administration of local anesthesia, three vertical incisions were performed using No. 15 blade mesial and distal the maxillary canine, and in the middle of the extraction site at a distance of 3-4 mm apically from the interdental papilla and 8-10 mm length. However, these incisions were made bilaterally with six buccal incisions (three at the upper right side and the same at the left one). Subsequently, a piezosurgery knife (BS1, Piezosurgery®, Mecrtron, Carasco, Italy) was inserted to accomplish the cortico-alveolar incisions with 3 mm depth (Figure 1). Ultimately, the incisions were not sutured and were simply covered with iodoform-impregnated gauze [24].

Regarding postoperative management, the patient was asked to follow a soft diet three days following the surgery to maintain good oral health, and no antibiotics were prescribed [25,26]. Moreover, in moderate or severe pain, the patient was allowed to take paracetamol (acetaminophen, 500 mg), provided that the questionnaire was filled out first.

LLLT Procedure

**Figure 2:**
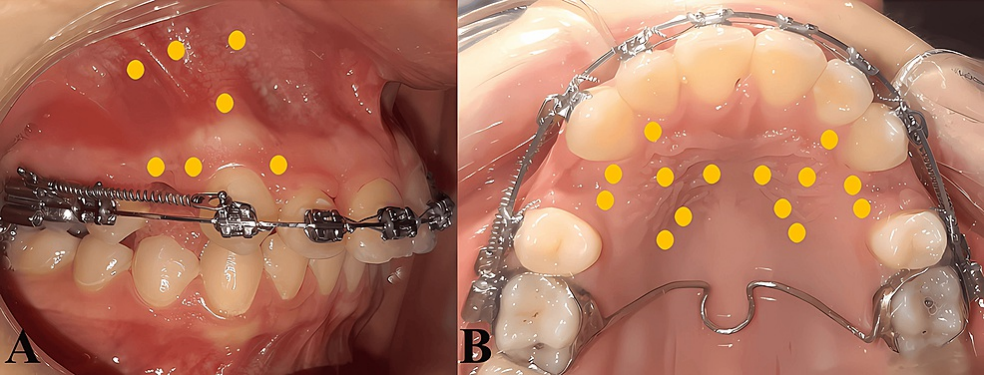
Low-level laser application points: A, the buccal side; B, the palatal side

LLLT was performed by the same principal investigator (DTA). The LLLT device used in this trial was a semiconductor diode laser of gallium aluminum arsenide (GaAlAs) (Mercury, Pioon, Hubei, China). It was operated in a continuous wave mode with a wavelength of 810 nm and a power output of 500 mW, energy (5) joules/point, and application time (10) seconds/point. During the LLLT application, irradiation was conducted by holding the laser tip perpendicular to the oral mucosa. To ensure irradiation of the entire area around the canines, LLLT was applied buccally on the root of the maxillary canine on both sides as follows: two doses of irradiation on the cervical third (one mesial and one distal), two doses of irradiation on the apical third (one mesial and one distal), and an irradiation dose on the middle third (in the middle of the root). Furthermore, two irradiation doses were applied cervically and apically in the middle of the first premolar extraction space on both the right and left sides (Figure 2). However, irradiations were conducted similarly on the palatal side. LLLT began at the beginning of maxillary canine retraction and was repeated on days 3, 7, 14, and 28 of the first month. Then, it was applied every two weeks until the end of the canine retraction phase.

Canine retraction

The canine retraction commenced immediately before the application of acceleration procedures. The upper canines were distalized in all patients until achieving a class I relationship via a 0.019 × 0.025 in. SS basal wire with closed-coil springs (NT3 closed coil, American Orthodontics, Sheboygan, Wis, USA) elongated from the hook of the canine brackets to the bands of the first molars. However, the applied force from the spring was set to generate 150g on each side. A force gauge (040-711-00; Dentaurum, Ispringen, Germany) was used to set the initial force of retraction and to ascertain the amount of force applied in each follow-up session (every two weeks) [27].

Outcome measures: questionnaires

A questionnaire containing questions about levels of pain, discomfort, swelling, and chewing difficulty was distributed to patients during the first month of canine retraction commencement at the following time points.

**Figure 3:**
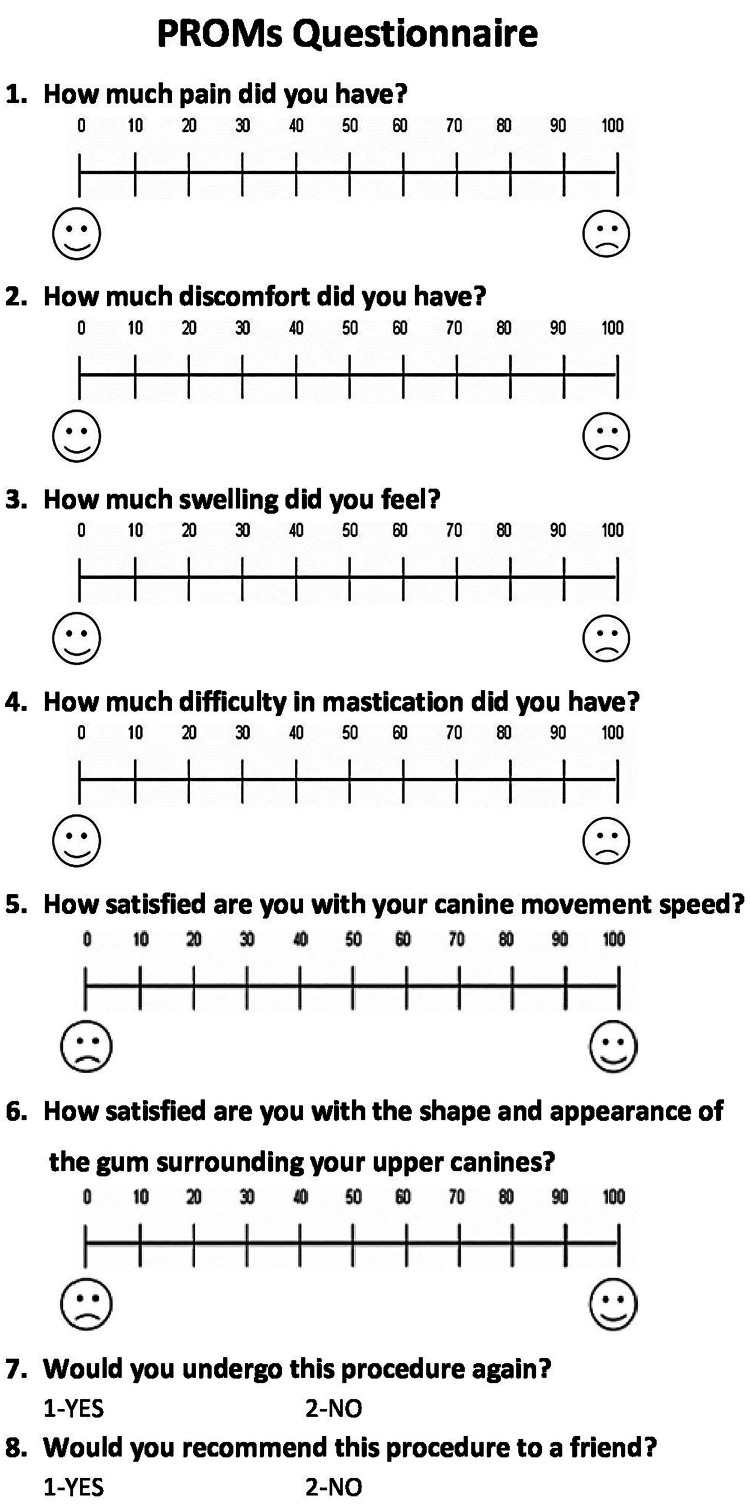
Questions given to the patients at five assessment time points. Questions 1-4 were given on the first day (T1), the third day (T2), the seventh day (T3), two weeks (T4), and four weeks (T5) after the onset of canine retraction, whereas questions 5-8 were administered at T5 only

At 24 hours (T1), after three days (T2), after seven days (T3), after 14 days (T4), and 28 days following the initiation of canine retraction (T5). Furthermore, at T5 (i.e., after 28 days), in addition to answering the previous questions, patients were asked to answer questions about satisfaction, acceptance, and consumption of analgesics (Figure 3). Regarding the pain assessment, the patients were guided that 0 meant 'no pain' and 100 meant 'the worst pain that can be felt'. The same method of reporting patients' experiences on this scale was clarified for the questions of discomfort, swelling, and chewing difficulty. However, the questions related to satisfaction, whether the canine speed or the shape and appearance of the gingiva surrounding it, were also answered using VAS, taking into account that 0 meant ‘no satisfaction’ and 100 meant ‘the best satisfaction’. On the other hand, ‘yes/no’ answered the questions related to the patient’s acceptance of the received procedure, whether they would recommend the procedure to a friend, and whether they took any type of painkillers.

Statistical analysis

All statistical analyses were executed using Statistical Product and Service Solutions (SPSS®, version 25.0; IBM Corp., Armonk, NY). Normality distribution was checked using the Shapiro-Wilk test. One-way ANOVA or its non-parametric alternative, Kruskal-Wallis, was applied to examine the difference between study groups. Independent t-test and its nonparametric alternative Mann-Whitney were applied for pairwise comparisons. Regarding the questions related to the patient's acceptance (undergoing the same procedure, recommendation of the procedure to a friend, and consumption of analgesics), the chi-square independence test was used to detect differences between the three groups. However, Friedman’s test was used to check the over-time changes in the studied variables. The results were considered significant when p-value <0.05. Furthermore, the Bonferroni correction was employed for the multiplicity of tests.

## Results

Baseline sample characteristics

**Figure 4:**
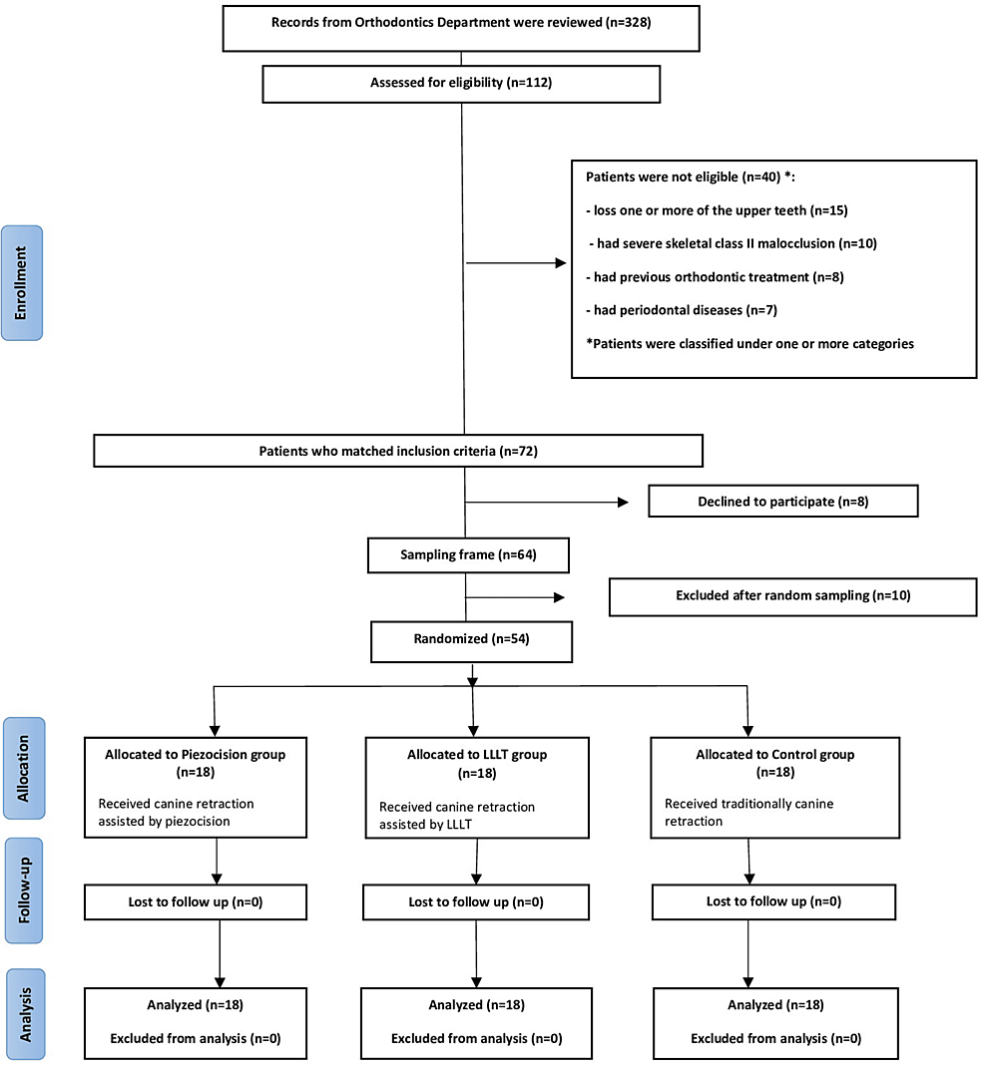
The Consolidated Standards of Reporting Trials (CONSORT) participants' flow diagram

Fifty-four patients (12 males, 42 females) at a mean age of 20.65±2.85 years were included in this trial. No dropout occurred during the trial, and all patients completed their questionnaires, as shown in Figure 4. The basic characteristics of the sample at the beginning of the treatment are displayed in Table 1. However, no significant differences were found between groups regarding gender and age (p=0.725, p=0.538, respectively; Table 1).

**Table 1:** Basic sample characteristics (gender and age) †Employing the Kruskal-Wallis test n: number of patients, SD: standard deviation, Min.: minimum, Max.: maximum, PG: Piezocision group, LLLTG: Low-level laser therapy group, CG: Control group *Employing the chi-square independence test

Group	Gender (n%)	P-value*	Mean Age ± SD	Min. Age	Max. Age	P-value†
PG (n=18)	Male 4 (22.2%)	0.725	21.16 ± 2.91	17.30	28.00	0.538
	Female 14 (77.8%)					
LLLTG (n=18)	Male 5 (27.8%)		20.22 ± 2.74	17.50	28.00	
	Female 13 (72.2%)					
CG (n=18)	Male 3 (16.7%) Female 15 (83.3%)		20.57 ± 2.99	17.20	28.00	
All sample (n=54)	Male 12 (22.2%)		20.65 ± 2.85	17.20	28.00	
	Female 42 (77.8%)					

Main findings: pain and discomfort

The greatest levels of pain and discomfort were recorded 24 hours after the initiation of canine retraction in all groups. However, the level was “mild” in the LLLTG (mean value: 10.05±6.70 mm for pain and mean value: 11.77±7.24 mm for discomfort; Table 2), while “mild to moderate” levels were recorded in both the PG and CG. The VAS values in the PG were the greatest, with a mean of 37.50±24.73 mm for pain and 39.66±25.46 mm for discomfort. Furthermore, in the CG, the mean VAS values for pain and discomfort were 20.22±8.49 mm and 24.16±9.85 mm, respectively. Then, the levels gradually decreased until the 28th day of retraction commencement, where they approached zero in the three groups.

**Table 2:** Descriptive statistics of the levels of pain, discomfort, swelling, chewing difficulty, and satisfaction reported by the patients, as well as the p-values of significance tests

Variables	Time	Piezocision group (n=18)			LLLT group (n=18)			Control group (n=18)			P- value	Significance
		Mean (SD)	Min	Max	Mean (SD)	Min	Max	Mean (SD)	Min	Max		
Pain†	T1	37.50 (24.73)	10.00	100.00	10.05 (6.70)	0.00	25.00	20.22 (8.49)	5.00	39.00	<0.001	***
	T2	32.83 (23.98)	0.00	85.00	7.27 (6.03)	0.00	20.00	15.38 (7.67)	5.00	34.00	<0.001	***
	T3	18.88 (13.77)	0.00	50.00	3.05 (3.97)	0.00	16.00	8.88 (4.57)	0.00	17.00	<0.001	***
	T4	4.72 (4.62)	0.00	15.00	0.61 (2.35)	0.00	10.00	2.55 (5.47)	0.00	20.00	<0.003	**
	T5	1.05 (1.62)	0.00	5.00	0.05 (0.23)	0.00	1.00	0.66 (2.35)	0.00	10.00	0.035	*
Discomfort†	T1	39.66 (25.46)	10.00	100.00	11.77 (7.24)	0.00	23.00	24.16 (9.85)	5.00	40.00	<0.001	***
	T2	31.33 (18.13)	10.00	77.00	8.22 (6.23)	0.00	20.00	16.27 (8.95)	0.00	35.00	<0.001‡	***
	T3	20.72 (9.99)	8.00	44.00	4.50 (5.09)	0.00	20.00	8.33 (6.10)	0.00	26.00	<0.001	***
	T4	6.00 (4.87)	0.00	18.00	1.11 (2.51)	0.00	10.00	4.00 (4.37)	0.00	16.00	0.002	**
	T5	0.33 (1.41)	0.00	6.00	0.27 (0.75)	0.00	3.00	0.44 (1.88)	0.00	8.00	0.484	NS
Swelling†	T1	25.16 (22.25)	0.00	90.00	0.05 (0.23)	0.00	1.00	0.05 (0.23)	0.00	1.00	<0.001	***
	T2	21.88 (22.61)	0.00	75.00	0.05 (0.23)	0.00	1.00	0.05 (0.23)	0.00	1.00	<0.001	***
	T3	3.33 (11.88)	0.00	50.00	0.05 (0.23)	0.00	1.00	0.05 (0.23)	0.00	1.00	0.721	NS
	T4	0.11 (0.32)	0.00	1.00	0.05 (0.23)	0.00	1.00	0.05 (0.23)	0.00	1.00	0.767	NS
	T5	0.11 (0.32)	0.00	1.00	0.11 (0.32)	0.00	1.00	0.05 (0.23)	0.00	1.00	0.805	NS
Chewing difficulty†	T1	33.88 (23.94)	0.00	80.00	13.27 (7.51)	3.00	32.00	20.33 (9.51)	0.00	35.00	0.001‡	***
	T2	29.61 (17.30)	0.00	64.00	9.44 (6.07)	2.00	25.00	16.11 (8.52)	0.00	30.00	<0.001‡	***
	T3	16.11 (12.24)	0.00	33.00	4.16 (4.43)	0.00	16.00	7.66 (6.54)	0.00	22.00	0.008	**
	T4	2.00 (3.44)	0.00	10.00	1.00 (2.16)	0.00	8.00	3.83 (4.19)	0.00	10.00	0.096	NS
	T5	0.05 (0.23)	0.00	1.00	0.11 (0.32)	0.00	1.00	0.11 (0.47)	0.00	2.00	0.789	NS
Satisfaction with canine speed †	T5	94.05 (7.84)	75.00	100.00	90.50 (6.27)	75.00	100.00	71.05 (11.57)	50.00	84.00	<0.001	***
Satisfaction with the canine gum appearance	T5	88.72 (10.42)	60.00	100.00	93.33 (5.16)	83.00	100.00	95.00 (7.06)	78.00	100.00	0.061	NS

The intergroup pairwise comparisons showed that pain and discomfort perception in the PG was significantly greater in comparison with the LLLTG at T1, T2, T3 (p<0.001; Table 3), and T4 (p=0.001). Moreover, the pain levels in the PG were significantly greater compared to that in the CG at T1, T2, and T3 (p=0.002, p=0.008, and p=0.009, respectively), whereas the discomfort levels were significantly greater at T2 and T3 (p=0.011 and p<0.001, respectively). On the other hand, the experienced pain levels in the CG were significantly greater compared to that in the LLLTG at T1, T2, and T3 (p=0.001, p=0.001, and p<0.001, respectively), whereas the discomfort levels were significantly greater at T1, T2, and T4 (p=0.001, p=0.010, and p=0.007, respectively).

**Table 3:** Results of the significance tests of pairwise comparisons between the evaluated time points for patient-reported outcomes in the three studied groups (n=54) †Employing the Mann-Whitney U test, ‡Employing Games-Howell n: number of patients, T1: after 24 hours of canine retraction commencement, T2: after 3 days, T3: after 7 days, T4: after 14 days, T5: after 28 days, LLLT: Low-level laser therapy *significant at p<0.05, ** significant at p< 0.017 with Bonferroni adjustment for the alpha level (0.05/3 = 0.017), NS: non-significant

Variable	Time	Pairwise comparison	Mean difference	p-value	Significance
Pain†	T1	Piezocision vs. LLLT	27.45	<0.001	***
		Piezocision vs. Control	17.28	0.002	**
		LLLT vs. Control	-10.17	0.001	**
	T2	Piezocision vs. LLLT	25.56	<0.001	***
		Piezocision vs. Control	17.45	0.008	**
		LLLT vs. Control	-8.11	0.001	**
	T3	Piezocision vs. LLLT	15.83	<0.001	***
		Piezocision vs. Control	10.00	0.009	**
		LLLT vs. Control	-5.83	<0.001	***
	T4	Piezocision vs. LLLT	4.11	0.001	**
		Piezocision vs. Control	2.17	0.035	*
		LLLT vs. Control	-1.94	0.342	NS
Discomfort†	T1	Piezocision vs. LLLT	27.89	<0.001	***
		Piezocision vs. Control	15.5	0.036	*
		LLLT vs. Control	-12.39	0.001	**
	T2	Piezocision vs. LLLT	23.11	<0.001‡	***
		Piezocision vs. Control	15.06	0.011‡	**
		LLLT vs. Control	-8.05	0.010‡	**
	T3	Piezocision vs. LLLT	16.22	<0.001	***
		Piezocision vs. Control	12.39	<0.001	***
		LLLT vs. Control	-3.83	0.021	*
	T4	Piezocision vs. LLLT	4.89	0.001	**
		Piezocision vs. Control	2.00	0.180	NS
		LLLT vs. Control	-2.89	0.007	**
Swelling†	T1	Piezocision vs. LLLT	25.11	<0.001	***
		Piezocision vs. Control	25.11	<0.001	***
		LLLT vs. Control	0.00	1.00	NS
	T2	Piezocision vs. LLLT	21.83	<0.001	***
		Piezocision vs. Control	21.83	<0.001	***
		LLLT vs. Control	0.00	1.00	NS
Chewing difficulty‡	T1	Piezocision vs. LLLT	20.61	0.006	**
		Piezocision vs. Control	13.55	0.088	NS
		LLLT vs. Control	-7.06	0.049	*
	T2	Piezocision vs. LLLT	20.17	<0.001	***
		Piezocision vs. Control	13.50	0.017	*
		LLLT vs. Control	-6.67	0.029	*
	T3	Piezocision vs. LLLT	11.95	0.008†	**
		Piezocision vs. Control	8.45	0.032†	*
		LLLT vs. Control	-3.50	0.084†	NS
Satisfaction with canine speed†	T5	Piezocision vs. LLLT	3.55	0.034	*
		Piezocision vs. Control	23.00	<0.001	***
		LLLT vs. Control	19.45	<0.001	***

Swelling and chewing difficulty

The perception of swelling was almost non-existent in the LLLTG and CG at all evaluation times (0.05±0.23). In comparison, the feeling of swelling was "mild to moderate" in the PG after 24 hours and three days of the canine retraction initiation (mean values: 5.16±22.25 mm and 21.88±22.61 mm, respectively), with statistically significant differences when compared with that of the LLLTG and CG at T1 and T2 (p<0.001). Thereafter, the swelling perception in the PG decreased to “mild” in the remaining evaluation times without any significant differences when compared with the other two groups.

Regarding chewing difficulty, the highest levels were recorded 24 hours after the canine retraction initiation in each of the three groups. However, these levels were “mild” in the LLLTG (mean value: 13.27±7.51 mm) and “mild to moderate” in both the PG (mean value: 33.88±23.94 mm) and CG (mean value: 20.33±9.51 mm). The levels gradually decreased until the 28th day of retraction commencement, when they approached zero. However, statistically significant differences between groups were found only when comparing PG with the LLLTG at the T1, T2, and T3 (p=0.006, p<0.001, and p=0.008, respectively) in favor of the PG.

Satisfaction, acceptance, and analgesic consumption

After four weeks of canine retraction commencement, the patients in the experimental groups (i.e., PG and LLLTG) reported "high" levels of satisfaction with canine speed (mean values: 94.05±7.84 mm and 90.50±6.27 mm, respectively), with no statistically significant difference between the two groups (p=0.034). However, the satisfaction levels with the canine speed in the CG were "moderate to high" (mean value: 71.05±11.57 mm), with statistically significant differences when compared with those in the experimental groups (p<0.001). On the other hand, satisfaction with the canine gum appearance at T5 was “high” in three groups, with no statistically significant difference (mean values: 88.72±10.42, 93.33±5.16, and 95.00±7.06 mm in the PG, LLLTG, and CG, respectively; p=0.061).

Regarding acceptance, only two patients (11.1%, Table 4) in the PG reported that they would not undergo the same procedure or recommend a friend to undergo it. However, the proportion difference between the three groups was insignificant (p=0.125). The amount of analgesic consumption was low in both PG and CG (11.1%) and did not differ significantly between the three groups (p=0.340).

**Table 4:** Descriptive statistics of the acceptance levels reported by the patients after 28 days of canine retraction commencement (T5), as well as the p-values of significance tests †Employing the chi-square independence test LLLT: Low-level laser therapy, n: number of patients, NS: there was no statistically significant difference at p>0.05

Question	Response	Piezocision group (n=18)		LLLT group (n=18)		Control group (n=18)		P-value†	Significance
		n	%	n	%	n	%		
Undergo the same procedure	Yes	16	88.9	18	100.00	18	100.00	0.125	NS
	No	2	11.1	0	0.00	0	0.00		
Recommend the procedure to a friend	Yes	16	88.9	18	100.00	18	100.00	0.125	NS
	No	2	11.1	0	0.00	0	0.00		

Changes over time within groups

Friedman’s test results showed that there were significant differences between the five assessment time points regarding the levels of pain (p<0.001), discomfort (p<0.001), and chewing difficulty (p<0.001) for the three groups. However, differences between evaluation times regarding the swelling levels were significant only in the PG (p <0.001).

Harms

During the entire trial duration, no significant harm occurred. None of the patients experienced post-surgical complications, such as hematomas, gingival recession, lower lip numbness, or any other short-term side effects. However, due to fear of undergoing the surgical procedure, one patient showed symptoms of dizziness and hypotension at the end of the procedure. The necessary first aid was performed, and the patient was monitored until it was confirmed that she had returned to normal condition. Subsequently, she was kept in touch to ensure she was healthy.

## Discussion

To our knowledge, this is the first RCT in the literature comparing a surgical technique versus a non-surgical one regarding PROMs during the retraction of the maxillary canines. The visual analog scale was used as a tool for PROM assessment because of its superiority over other scales [12] and its adoption in previous trials [28,29]. In addition, this scale is easy to understand for most patients; its sensitivity to small differences is good and has good reproducibility [30]. Patients' responses were recorded 24 hours after the upper canine retraction initiation in the three groups. However, it was not taken immediately after applying the retraction springs so that the analgesia resulting from the local anesthesia used during the surgical intervention in the piezocision group would not affect the study results.

Our study showed that the levels of pain and discomfort were significantly lower in the LLLTG during the first two weeks following the onset of canine retraction compared to the other two groups. This can be attributed to the analgesic effect associated with the application of LLLT, which can be explained by two different mechanisms: the first is that LLLT stimulates the production of beta-endorphin, which leads to the production of metabolites that interact with the receptors of pain [31]. The second mechanism relies on the “sodium-potassium pump” to block the localized painful impulse transmission [31]. This was consistent with the results of previous studies, which found that LLLT was effective in relieving pain in cases of upper canine retraction [13,15].

On the other hand, when comparing the piezocision with the control group, pain levels were significantly greater in the piezocision group during the first week of the retraction initiation, while the levels of discomfort in this group were significantly greater three and seven days after the retraction commencement. This can be explained by the injury to the gingival soft tissues and alveolar bone during the piezocision application. However, the levels of pain and discomfort in the piezocision group were “mild to moderate” on the day after the surgical intervention and decreased on the following seven days, which was in line with the results of the previous systematic reviews [19,32]. This could be explained by the rapid healing associated with the application of piezocision [33,34].

Mild to moderate levels of swelling perception were reported by the patients of the piezocision group after 24 hours and three days of the surgical intervention, with significant differences when compared with the other two groups. This may be attributed to the edema associated with the surgical trauma caused by such procedures. Thereafter, no significant difference was found between the three groups regarding swelling perception, which could be attributed to the conservative nature and minimal trauma accompanying the application of this technique. These findings disagreed with Alfawal et al. [34], who found that swelling levels on the piezocision side were only significantly greater 24 hours after the intervention than on the control side. This difference can be attributed to the fact that their study was a split-mouth design, so the feeling of the patient recorded is only for one-half of the face and cannot reflect the full truth of what would be the case if the procedure was applied from both sides. In addition, in our study, three cortical cuts were applied on each side (i.e., six cuts on both sides), but in their studies, two cortical cuts were applied unilaterally.

Chewing difficulty perception was significantly greater in the piezocision group during the first week of retraction commencement (ranging from 33.88 to 16.11 mm) than in the LLLTG (ranging from 13.27 to 4.16 mm). This could be related to the post-piezocision pain and discomfort that patients experienced. Unfortunately, the results of chewing difficulty related to the acceleration interventions in the current trial cannot be compared with the findings of others due to the absence of similar trials.

In the current study, they were recorded one month after the canine retraction initiation and accelerated intervention applications to accurately assess the levels of satisfaction with the orthodontic procedure performed. The piezocision group and the LLLTG showed significantly greater levels of canine speed satisfaction (mean values: 94.05 mm and 90.50 mm, respectively) than the control group (mean value: 71.05 mm). The higher satisfaction levels in the experimental groups can be attributed to a greater rate of OTM. Thus, the patients noticed a clear movement in their upper canines, reflecting their satisfaction levels positively. On the other hand, levels of satisfaction with the shape and appearance of the gums surrounding the upper canines were high in the three groups. Although the levels of satisfaction in the PG (mean value: 88.72 mm) were lower than those in the LLLTG (mean value: 93.33 mm) and control one (mean value: 95.00 mm), they were at high values, and this can be attributed to the rapid recovery accompanying the application of this technique. However, when reviewing the literature, no previous studies have investigated the levels of satisfaction with the speed of the upper canines and the shape and appearance of the surrounding gums after the application of piezocision and LLLT, with which to compare the results of the current study.

Patients reported high acceptance levels of the applied treatment procedure, as the percentage in the LLLT and conventional retraction groups was 100% and 88.9% in the piezocision group, respectively, with negligible differences between the three groups. This was consistent with the findings of previous studies [28,34], which reported that patients' acceptance of the piezocision technique and the proportion of recommendations of this technique to friends were high. After we reviewed the literature, we found that the acceptance accompanied by the LLLT application in the context of maxillary canine retraction was not addressed in any of the previous relevant trials. Hence, it was impossible to compare with any previously published results.

Limitations

The current study had some limitations. PROM assessment was not conducted daily during the first week of implementing the acceleratory interventions. Furthermore, the follow-up duration was only a month from the commencement of upper canine retraction, while the retraction force was applied every two to three weeks till the end of the canine retraction. Therefore, this trial did not assess the perception of pain and discomfort that may be repeated in every activation session. Additionally, the effect of gender on the evaluation of the PROMs studied in this research was not detected. Finally, this research did not study the difficulty of swallowing and limitation in jaw movement associated with the application of piezocision and LLLT.

Generalizability

This study was a single-center RCT that included patients with a specific type of malocclusion (i.e., Class II division 1 malocclusion) with a specific age group (17-28 years). As a result, the current trial's findings can only be generalized to orthodontic patients with conditions similar to those in this trial.

## Conclusions

The LLLT-assisted canine retraction was associated with significantly lower levels of pain and discomfort during the first two weeks of tooth movement compared to the piezocision-assisted and conventional retraction groups. On the other hand, piezocision-assisted retraction was associated with significantly greater pain and discomfort during the first week of tooth movement compared to the conventional retraction group. Patients’ perception of swelling following piezocision was at mild-to-moderate levels in the first three days of the procedure. The LLLT-assisted canine retraction was associated with lower levels of chewing difficulty during the first week of procedure application and retraction compared with piezocision. Patient satisfaction and acceptance during the first month of canine retraction were high with piezocision and LLLT.
